# The relationship between carbohydrate intake and sleep patterns

**DOI:** 10.3389/fnut.2024.1491999

**Published:** 2024-12-04

**Authors:** Yan Zhao, Hailong Guo

**Affiliations:** ^1^Faculty of Basic Medical Sciences, Chongqing Medical and Pharmaceutical College, Chongqing, China; ^2^Health Management Center, The First Branch, The First Affiliated Hospital of Chongqing Medical University, Chongqing, China

**Keywords:** carbohydrate quality, carbohydrate quantity, sleep duration, sleepy, sleep patterns, snoring

## Abstract

**Background:**

A healthy dietary habit may contribute to good sleep quality. The present study investigates the correlation between the quality and quantity of daily carbohydrate consumption and poor sleep patterns.

**Methods:**

The exposures of interest included low-and high-quality carbohydrate consumption and total daily carbohydrate consumption. Subjects were classified into four different carbohydrate consumption patterns: Pattern 1 was characterized by high-quality carbohydrates below the median and low-quality carbohydrates above the median; Pattern 2 included both high-and low-quality carbohydrates below the median; Pattern 3 was defined as high-and low-quality carbohydrates above the median; Pattern 4 referred to high-quality carbohydrates above the median and low-quality carbohydrates below the median. The comprehensive sleep patterns included three different sleep behaviors: sleep duration, daytime sleepiness, and snoring, which were used to score sleep patterns. A score ranging from 0 to 1 was classified as having a healthy sleep pattern, while a score between 2 and 3 showed poor sleep patterns. Survey-weighted multivariable logistic regression analyses were adopted.

**Results:**

In the multivariate analysis, individuals who consumed more high-quality carbohydrates were linked to a decreased likelihood of experiencing poor sleep patterns [odds ratio (OR) 0.71; 95% confidence interval (CI) 0.62–0.81], while increased consumption of low-quality carbohydrates (OR 1.39; 95%CI 1.20–1.61) and total daily carbohydrates (OR 1.31; 95%CI 1.10–1.57) was related to an elevated risk of poor sleep patterns. Participants who adhered to carbohydrate intake pattern 4 exhibited a 36% lower risk of poor sleep patterns than those who followed carbohydrate intake pattern 1 (OR 0.64; 95%CI 0.56–0.74). There was a positive correlation between elevated added sugar consumption and an increased probability of developing poor sleep patterns. In contrast, an elevated intake of whole grains, fruits, or non-starchy vegetables was related to a decreased likelihood of experiencing poor sleep patterns.

**Conclusion:**

The increased consumption of low-quality carbohydrates may heighten the susceptibility to poor sleep patterns, whereas the increased consumption of high-quality carbohydrates may mitigate the risk of developing poor sleep patterns.

## Introduction

Sleeping less than 7 h per day is commonly referred to as short sleep duration. Recent studies have shown that over 30% of American adults are experiencing insufficient sleep duration, marking a 15% increase since 2004 (1, 2). Insufficient sleep, whether in terms of quality or quantity, has been proven to be associated with various diseases such as diabetes, obesity, stroke, cardiovascular disease, and cancer (3–7). It has been shown, however, that a healthy sleep pattern, characterized by early bedtime, normal sleep duration, the absence of insomnia, snoring, and daytime sleepiness, reduces the risk of cardiovascular disease and stroke (8). As a result, sleep health has emerged as a prominent public concern, garnering heightened awareness and attention from individuals (9).

Increasing evidence suggests a reciprocal relationship between sleep and dietary habits. Research indicated that short sleepers tended to consume higher amounts of energy, particularly from snacks and sugary foods, compared to normal sleepers (10, 11). Additionally, individuals with restricted sleep tended to have higher calorie consumption than those with adequate sleep (12). On the other hand, consumption of high-energy drinks, confectionary, and noodles has been linked to worse sleep quality (13, 14). Furthermore, a high glycemic index diet might increase the risk of insomnia in postmenopausal women (15). These findings indicated a complex relationship between dietary consumption and sleep quality, warranting additional investigation. Carbohydrates, as the primary macronutrient in the diet, contribute more than half of total energy intake. Effective management of energy levels hinges on regulating carbohydrate consumption. Dietary carbohydrate quality can be divided into high-quality carbohydrates composed of whole grains, fruits, vegetables, and legumes, as well as low-quality carbohydrates composed of added sugars, refined grains, fruit juices, and starchy vegetables (16). However, few studies focused solely on carbohydrates and exploring their correlation with sleep.

Given the potential for carbohydrates to be more readily modified compared to sleep quality, this study posits the quality and quantity of carbohydrates as the independent variable and sleep quality as the dependent variable. This study aims to investigate the correlation between high-and low-quality carbohydrate consumption, total daily carbohydrate consumption, and poor sleep patterns utilizing data from the National Health and Nutrition Examination Survey (NHANES).

## Materials and methods

### Study population

The NHANES survey is an ongoing nationally cross-sectional study that employed complex, stratified, and multistage probability sampling methodologies to provide the health and nutritional condition of adults and children in the United States. For details, please refer to the official website.^1^

This study involved four NHANES cycles (2005–2006, 2007–2008, 2015–2016, and 2017–2018) and included a total of 39,722 individuals. After excluding individuals under 20 years of age, pregnant females, those with missing two-day dietary recall data, and incomplete sleep questionnaires, a final sample of 17,366 participants was analyzed (Supplementary Figure S1).

### Exposures

Dietary data for each individual was collected from the average of two 24-h dietary recall interviews. The first dietary recall was administered in person by trained interviewers in the NHANES Mobile Examination Center (MEC) and the second was completed by trained interviewers via telephone 3–10 days after the MEC interview. Participants were asked to report the types and amounts of foods and beverages (including all types of water) consumed within the 24 h before the interview. Both 24-h dietary recalls were collected using the computerized US Department of Agriculture (USDA) Automated Multiple-Pass Method (17). Dietary information was obtained from the Food Patterns Equivalents Database (FPED), which standardizes the foods and beverages reported in the NHANES Database into the 37 USDA Food Patterns components. Food patterns are assessed using standardized measurements, including cup equivalents for Fruit, Vegetables, and Dairy; ounce equivalents for Grains and Protein Foods; teaspoon equivalents for Added Sugars; gram equivalents for Solid Fats, Oils, and Alcoholic Drinks. The variables of interest are the quality and quantity of daily carbohydrate consumption. This study assessed the quality of daily carbohydrate consumption by distinguishing between low-quality and high-quality carbohydrates. The calculation methodology was derived from a prior study (16). The total daily high-quality carbohydrate consumption was determined by adding the intake of whole grains, legumes, whole fruits, and non-starchy vegetables together. Conversely, the total daily low-quality carbohydrate consumption was the sum of the intake of refined grains, fruit juices, starchy vegetables, and added sugars (Supplementary Table S1). To better investigate the association between dietary carbohydrates and sleep patterns, subjects were divided into four different carbohydrate consumption patterns according to median values relative to their high-and low-quality carbohydrate consumption. Pattern 1 denoted high-quality carbohydrates below the median and low-quality carbohydrates above the median. Pattern 2 included both high-and low-quality carbohydrates below the median. Pattern 3 was defined as high-and low-quality carbohydrates above the median. Pattern 4 referred to high-quality carbohydrates above the median and low-quality carbohydrates below the median. The total daily quantity of carbohydrates was calculated by adding high-and low-quality carbohydrates.

### Outcomes

Data on sleep behaviors was acquired from the Sleep Disorders (SLQ) module. Each participant was asked at home by trained interviewers using the Computer-Assisted Personal Interview system. Sleep duration was determined by responding to the question, “How much sleep do you typically get at night on weekdays or workdays?” Sleep duration was categorized into two groups: 1 point for sleep duration greater than 9 h (long) or less than 7 h (short), and 0 points for sleep duration within the normal range of 7–9 h (18). Information on snoring symptoms was collected through the question, “How frequently do you snore?” Participants reporting never or rare occurrences of snoring were assigned a score of 0, while those reporting occasional or frequent snoring were assigned a score of 1. Daytime sleepiness symptoms were evaluated by asking, “How often do you feel excessively sleepy during the day?” Participants reporting sleepiness four or fewer times per month were assigned a zero score, while those reporting more than four times per month were assigned a one score (19, 20). The above three sleep behaviors (sleep duration, overly sleepy, and snoring) were used to calculate sleep scores. According to prior studies (19, 20), individuals with a sleep score of 0–1 were classified as having healthy sleep patterns. In contrast, those with a sleep score ranging from 2 to 3 were categorized as showing poor sleep patterns.

### Covariates

The baseline information gathered for this study encompassed age, sex, race/ethnicity, body mass index (BMI), education level (less than high school, high school graduates, and above high school), marital status, family income (<1.3, 1.3–3.5 and ≥3.5), smoking habits (never, former, and current), alcohol status (none, mild, moderate, heavy, and former), physical activity, disease history (hypertension, diabetes, hyperlipidemia, and depression), and other dietary information (total energy intake, saturated fatty acids, total fat, and dietary fiber). Marital status was classified as either with a partner (including married and living with a partner) or without a partner (including individuals who were never married, separated, divorced, or widowed). Based on weekly metabolic equivalents (METs), physical activity was classified as low (<7.5 METs-h/wk), moderate (7.5–30 METs-h/wk), and high (>30 METs-h/wk). The identification of hypertension, diabetes, and hyperlipidemia in participants aligns with findings from our prior study (21). Depression symptoms were evaluated through the Patient Health Questionnaire, and a score of 10 or more was diagnosed with depression.

### Statistical analysis

Following the NHANES analysis guidelines, the appropriate survey weight was determined by the weight of the smallest subpopulation within the variable of interest.^2^ Therefore, we used interview weights (wtint2yr) from four cycles divided by 4 and adjusted for variance in R 4.2.2 to explain the complex sampling design of NHANES. Baseline characteristics were shown as means ± standard error for continuous variables and percentages for categorical variables. Student *t*-tests were used to compare continuous data, while Chi-square tests were employed for categorical data analysis.

Survey-weighted multivariable logistic regression analyses were adopted to examine the association between quantity and quality of daily carbohydrate consumption and sleep patterns. Initially, we examined the relationship between four carbohydrate intake patterns, daily high-and low-quality carbohydrate consumption, total daily carbohydrate intake, and sleep patterns. Subsequently, we also explored their relationship with three sleep behaviors (sleep duration, snoring, and daytime sleepiness). Three models were developed to control for potential confounding variables. Model I served as the unadjusted model, while Model II included adjustments for age, gender, and race. Model III, the fully adjusted model, accounted for all covariates. We also employed the multivariable logistic regression model based on restricted cubic splines (RCS) to investigate the potential non-linear or linear correlation between daily carbohydrate intake and sleep patterns. Further, survey-weighted multivariable logistic regression analyses were conducted to elucidate the association between the consumption of daily specific foods and sleep patterns and behaviors.

Beyond the analyses above, several sensitivity analyses were also carried out to enhance the validity of our results. Firstly, considering the association between HEI-2015 and both carbohydrate intake and sleep disorders (22), adjustments were made for HEI-2015 in Model III. Likewise, given that depression was closely related to carbohydrate intake and sleep disorders (23), depression was introduced into Model III. Besides, given the correlation between carbohydrate intake and dietary factors, we further adjusted for saturated fatty acids, total fat, and dietary fiber. Finally, individuals with implausible energy intake levels falling below 500 kcal or exceeding 6,000 kcal per day were excluded from the analysis to enhance the examination of the interplay between the quality and quantity of carbohydrate intake and sleep patterns (24). *p-*values < 0.05 were considered statistically significant.

## Results

Table 1 presents the baseline features of study populations with different sleep patterns. Individuals with poor sleep patterns showed higher daily consumption of total carbohydrates and low-quality carbohydrates, along with a lower consumption of high-quality carbohydrates, compared to their counterparts with healthy sleep patterns (all *p* < 0.001). Additionally, a greater percentage of individuals with unhealthy sleep patterns adhered to carbohydrate intake pattern 1, while a smaller percentage adhered to carbohydrate intake pattern 4 (*p* < 0.001). Subjects with poor sleep patterns exhibited older age, a higher percentage of males, Black, and current smokers, tended to exercise less, and higher levels of BMI, total energy intake, as well as increased rates of hypertension, diabetes, and hyperlipidemia. Conversely, participants with healthy sleep patterns displayed higher levels of education and family income, and a higher percentage of participants who did not drink alcohol to moderate alcohol. Participants who adhered to carbohydrate intake pattern 4 exhibited a lower prevalence of snoring and excessive daytime sleepiness, and a higher proportion of participants with normal sleep duration, suggesting a healthier sleep pattern compared to those following carbohydrate intake pattern 1 (Table 2, all *p* < 0.001).

**Table 1:** Characteristics of the study population based on healthy sleep and unhealthy sleep patterns.

Study variables	Total	Healthy sleep	Poor sleep	*P-*value
	*N* = 17,366	*N* = 11,962	*N* = 5,404	
Age (years)	47.52 (0.36)	45.74 (0.50)	48.20 (0.38)	<0.001
Sex (%)				<0.001
Male	49.13	47.72	52.53	
Female	50.87	52.28	47.47	
Ethnicity (%)				<0.001
Black	11.05	9.86	13.93	
White	67.47	68.20	65.72	
Mexican	8.32	8.46	7.97	
Other	13.16	13.48	12.38	
Body mass index (kg/m^2^)	29.30 (0.15)	27.22 (0.20)	30.11 (0.15)	<0.001
Education level (%)				<0.001
Less than high school	14.58	13.91	16.18	
High school graduates	24.93	23.86	27.54	
Above high school	60.47	62.21	56.26	
Unknown	0.02	0.02	0.03	
Marital status (%)				0.140
With partner	34.72	34.01	36.43	
Without partner	65.25	65.95	63.54	
Unknown	0.03	0.03	0.03	
Family poverty-to-income ratio (%)				<0.001
< 1.3	17.97	16.41	21.75	
1.3–3.5	33.33	32.92	34.34	
≥ 3.5	41.75	43.58	37.33	
Unknown	6.94	7.09	6.58	
Smoking status (%)				<0.001
Never	54.69	57.70	47.41	
Former	24.65	24.66	24.65	
Current	20.60	17.61	27.85	
Unknown	0.05	0.03	0.10	
Alcohol consumption (%)				<0.001
None	9.71	10.27	8.36	
Mild	34.08	35.26	31.21	
Moderate	16.54	17.14	15.08	
Heavy	19.88	18.84	22.41	
Former	10.97	9.83	13.73	
Unknown	8.82	8.66	9.21	
Physical activity (%)				0.010
Low	45.00	42.61	45.98	
Moderate	27.42	26.99	27.60	
High	27.58	30.39	26.41	
Hypertension (%)	37.76	34.47	45.70	<0.001
Diabetes (%)	14.03	12.17	18.51	<0.001
Hyperlipidemia (%)	68.19	66.67	71.87	<0.001
Total energy intake (kcal/day)	2145.85 (13.58)	2093.28 (24.28)	2166.16 (15.60)	0.011
Total carbohydrates (servings/day)	25.39 (0.27)	23.89 (0.40)	25.97 (0.29)	<0.001
High-quality carbohydrate (servings/day)	2.75 (0.06)	3.03 (0.08)	2.64 (0.05)	<0.001
Low-quality carbohydrate (servings/day)	22.64 (0.29)	20.87 (0.42)	23.33 (0.32)	<0.001
Carbohydrate intake patterns (%)				<0.001
Pattern 1	26.63	21.90	28.48	
Pattern 2	21.85	21.36	22.04	
Pattern 3	23.55	23.33	23.63	
Pattern 4	27.98	33.40	25.86	

**Table 2:** The distribution of sleep pattern and behaviors by four carbohydrate intake patterns.

Study variables	Carbohydrate intake patterns					*P-*value
	Total	Pattern 1	Pattern 2	Pattern 3	Pattern 4	
	*N* = 17,366	*N* = 4,679	*N* = 4,006	*N* = 4,004	*N* = 4,677	
Sleep duration (%)						<0.001
Short or long sleep	35.01	40.23^b,c,d^	38.64^a,c,d^	33.51^a,b,d^	28.48^a,b,c^	
Sleepy (%)	23.23	27.09^b,c,d^	23.79^a,d^	23.27^a,d^	19.10^a,b,c^	<0.001
Snoring (%)	48.78	52.16^b,d^	45.57^a,c^	52.23^b,d^	45.15^a,c^	<0.001
Sleep patterns (%)						<0.001
Poor sleep	29.27	35.35^b,c,d^	29.96^a,d^	30.44^a,d^	21.96^a,b,c^	

^aRefers to comparing with Pattern 1, adjusted p < 0.05.^

^bRefers to comparing with Pattern 2, adjusted P < 0.05.^

^cRefers to comparing with Pattern 2, adjusted P < 0.05.^

^dRefers to comparing with Pattern 2, adjusted p < 0.05.^

Table 3 displays the results of the three multivariable logistic regression models used to examine the association between the quality and quantity of daily carbohydrate intake and the risk of experiencing poor sleep patterns. After accounting for all confounding variables, we observed the participants in the highest tertile of total daily carbohydrate and low-quality carbohydrate intake had a 31 and 39% higher risk of poor sleep patterns, respectively, compared to those in the lowest tertile. Conversely, individuals in the highest tertile of high-quality carbohydrate intake exhibited a 29% lower risk of unhealthy sleep patterns. Similarly, there was a 36% reduction in the risk of unhealthy sleep patterns in participants who adhered to carbohydrate intake pattern 4 relative to those who followed carbohydrate intake pattern 1.

**Table 3:** Associations of daily carbohydrate consumption quantity and quality with sleep patterns.

	Model I		Model II		Model III	
	OR (95% CI)	*P*	OR (95% CI)	*P*	OR (95% CI)	*P*
Carbohydrate intake patterns						
Pattern 1	Reference		Reference		Reference	
Pattern 2	0.78 (0.69, 0.89)	<0.001	0.81 (0.71, 0.94)	0.005	0.87 (0.73, 1.03)	0.100
Pattern 3	0.80 (0.70, 0.91)	<0.001	0.81 (0.71, 0.93)	0.003	0.92 (0.79, 1.07)	0.250
Pattern 4	0.51 (0.46, 0.58)	<0.001	0.55 (0.48, 0.63)	<0.001	0.64 (0.56, 0.74)	<0.001
*P* for trend	<0.001		<0.001		<0.001	
High-quality carbohydrate						
T1	Reference		Reference		Reference	
T2	0.78 (0.69, 0.88)	<0.001	0.80 (0.71, 0.91)	<0.001	0.87 (0.75, 0.99)	0.049
T3	0.61 (0.54, 0.68)	<0.001	0.63 (0.56, 0.70)	<0.001	0.71 (0.62, 0.81)	<0.001
*P* for trend	<0.001		<0.001		<0.001	
Low-quality carbohydrate						
T1	Reference		Reference		Reference	
T2	1.12 (0.98, 1.29)	0.090	1.11 (0.97, 1.27)	0.140	1.15 (0.99, 1.33)	0.070
T3	1.55 (1.39, 1.73)	<0.001	1.46 (1.29, 1.66)	<0.001	1.39 (1.20, 1.61)	<0.001
*P* for trend	<0.001		<0.001		<0.001	
Total carbohydrates						
T1	Reference		Reference		Reference	
T2	1.12 (0.96, 1.31)	0.130	1.11 (0.95, 1.30)	0.190	1.13 (0.95, 1.35)	0.150
T3	1.47 (1.29, 1.67)	<0.001	1.38 (1.19, 1.60)	<0.001	1.31 (1.10, 1.57)	0.004
*P* for trend	<0.001		<0.001		0.004	

OR, odds ratio; 95% CI, 95% confidence interval.

Model I was the crude model.

Model II adjusted for age, sex, and race/ethnicity.

Model III adjusted for model II+ education level, marital status, family poverty-to-income ratio, smoking status, alcohol consumption, physical activity, BMI, total energy intake, hypertension, diabetes, and hyperlipidemia.

**Figure 1:**
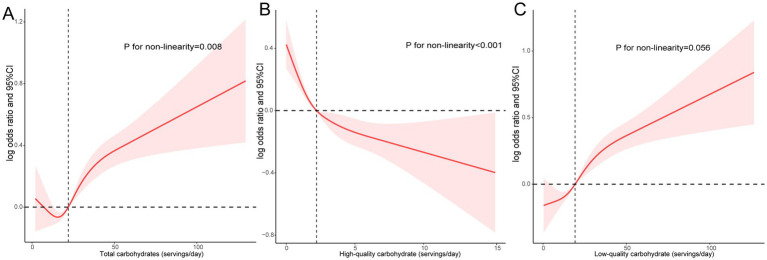
A dose–response association between daily carbohydrate consumption and sleep patterns. Associations between total **(A)**, high-quality **(B)**, and low-quality carbohydrate intake **(C)** with poor sleep patterns.

The study employed RCS to explore the dose–response correlation between daily carbohydrate consumption and sleep patterns (Figure 1). Results indicated that there was a U-shaped relationship between the total daily quantity of carbohydrates and unhealthy sleep patterns (*P* for non-linear = 0.008, Figure 1A). In addition, high-quality carbohydrate intake was non-linear and negatively associated with unhealthy sleep patterns (*P* for non-linear <0.001, Figure 1B). In contrast, a linear and positive relationship was observed between low-quality carbohydrate intake and poor sleep patterns (*P* for non-linear = 0.056, Figure 1C).

Table 4 further depicts the relationship between daily carbohydrate consumption quantity and quality with sleep behaviors. In the multivariate analysis, individuals adhering to carbohydrate intake pattern 4 demonstrated a 24% reduced risk of experiencing abnormal sleep duration, a 14% reduced risk of snoring, and a 31% reduced risk of daytime sleepiness, compared to those following carbohydrate intake pattern 1. Following adjustment for multiple variables, individuals in the T3 group consuming high-quality carbohydrates were found to have decreased odds of experiencing abnormal sleep duration and daytime sleepiness compared to those in the T1 group. In the multivariate model, participants who consumed the highest low-quality carbohydrate exhibited an 18% increased risk of experiencing too long or short sleep, a 24% elevated risk of snoring, and a 34% elevated risk of daytime sleepiness, compared to those in the lowest tertile of low-quality carbohydrate consumption. Similarly, individuals with the highest daily total carbohydrate consumption exhibited a higher risk of experiencing snoring and daytime sleepiness than those with the lowest consumption. Additionally, no significant correlation was observed between daily total carbohydrate consumption and abnormal sleep duration, nor was there a significant correlation between high-quality carbohydrate intake and snoring.

**Table 4:** Association of daily carbohydrate consumption quantity and quality with sleep behaviors.

	Sleep duration		Snore		Sleepy	
	Unadjusted model	Adjusted model	Unadjusted model	Adjusted model	Unadjusted model	Adjusted model
	OR (95% CI)	OR (95% CI)	OR (95% CI)	OR (95% CI)	OR (95% CI)	OR (95% CI)
Carbohydrate intake patterns						
Pattern 1	Reference	Reference	Reference	Reference	Reference	Reference
Pattern 2	0.94 (0.84, 1.04)	0.99 (0.86, 1.13)	0.77 (0.69, 0.85)^#^	0.85 (0.74, 0.98)^*^	0.84 (0.71, 0.99)^*^	0.86 (0.71, 1.04)
Pattern 3	0.75 (0.67, 0.84)^#^	0.91 (0.80, 1.04)	1.00 (0.90, 1.12)	1.01 (0.88, 1.16)	0.82 (0.69, 0.97)^*^	0.91 (0.75, 1.09)
Pattern 4	0.59 (0.53, 0.67)^#^	0.76 (0.66, 0.87)^#^	0.75 (0.68, 0.84)^#^	0.86 (0.76, 0.98)^*^	0.64 (0.56, 0.72)^#^	0.69 (0.59, 0.81)^#^
*P* for trend	<0.001	<0.001	<0.001	0.180	<0.001	<0.001
High-quality carbohydrate						
T1	Reference	Reference	Reference	Reference	Reference	Reference
T2	0.72 (0.64, 0.80)^#^	0.84 (0.74, 0.96)^*^	1.09 (0.98, 1.22)	1.10 (0.98, 1.25)	0.82 (0.73, 0.91)^#^	0.86 (0.76, 0.98)^*^
T3	0.59 (0.53, 0.65)^#^	0.76 (0.68, 0.85)^#^	0.91 (0.82, 1.01)	0.90 (0.79, 1.02)	0.71 (0.63, 0.80)^#^	0.78 (0.67, 0.91)^†^
*P* for trend	<0.001	<0.001	0.060	0.070	<0.001	0.002
Low-quality carbohydrate						
T1	Reference	Reference	Reference	Reference	Reference	Reference
T2	0.97 (0.86, 1.09)	0.98 (0.87, 1.11)	1.20 (1.08, 1.35)^†^	1.21 (1.06, 1.37)^*^	1.14 (0.98, 1.33)	1.18 (1.00, 1.39)^*^
T3	1.26 (1.15, 1.38)^#^	1.18 (1.04, 1.33)^*^	1.42 (1.30, 1.56)^#^	1.24 (1.10, 1.41)^†^	1.32 (1.14, 1.53)^#^	1.34 (1.11, 1.60)^†^
*P* for trend	<0.001	0.010	<0.001	<0.001	<0.001	0.003
Total carbohydrates						
T1	Reference	Reference	Reference	Reference	Reference	Reference
T2	0.90 (0.80, 1.02)	0.93 (0.82, 1.05)	1.18 (1.06, 1.32)^†^	1.16 (1.01, 1.33)^*^	1.12 (0.97, 1.30)	1.15 (0.98, 1.35)
T3	1.15 (1.04, 1.27)^*^	1.10 (0.96, 1.26)	1.41 (1.29, 1.55)^#^	1.22 (1.07, 1.39)^†^	1.27 (1.10, 1.48)^†^	1.29 (1.07, 1.55)^*^
*P* for trend	0.010	0.200	<0.001	0.003	0.002	0.010

OR, odds ratio; 95% CI, 95% confidence interval. *P < 0.05, ^†^*p* < 0.01, ^#^*p* < 0.001.

Adjusted model was adjusted for age, sex, race/ethnicity, education level, marital status, family poverty-to-income ratio, smoking status, alcohol consumption, physical activity, BMI, total energy intake, hypertension, diabetes, and hyperlipidemia.

Table 5 presents the relationship of specific food consumption with sleep patterns and behaviors using multivariable logistic regression models. Participants who consumed more added sugar daily were linked to a higher risk of experiencing poor sleep patterns, abnormal sleep duration, snoring, and daytime sleepiness. Besides, increased consumption of starchy vegetables was linked to a higher probability of experiencing daytime sleepiness. In contrast, for each 1-unit increase in non-starchy vegetable consumption, there was a 12% reduced likelihood of poor sleep patterns, a 7% reduced likelihood of abnormal sleep duration, and a 12% reduced likelihood of daytime sleepiness. Additionally, each unit increase in whole grains and fruit consumption was related to a reduced risk of experiencing abnormal sleep duration and poor sleep patterns. A negative correlation was observed between a one-unit increment in legume consumption and the risk of experiencing daytime sleepiness.

**Table 5:** Association of specific foods consumption with sleep pattern and behaviors.

Food	Sleep pattern	Sleep duration	Snore	Sleepy
	OR (95% CI)	OR (95% CI)	OR (95% CI)	OR (95% CI)
Whole grains	0.95 (0.91, 0.99)^*^	0.95 (0.91, 0.99)^*^	0.98 (0.94, 1.02)	0.96 (0.91, 1.01)
Legumes	0.86 (0.73, 1.01)	0.94 (0.78, 1.13)	1.02 (0.86, 1.21)	0.83 (0.70, 0.99)^*^
Whole fruits	0.91 (0.86, 0.97)^*^	0.92 (0.87, 0.98)^*^	0.97 (0.92, 1.02)	0.96 (0.89, 1.04)
Non-starchy vegetables	0.88 (0.83, 0.94)^#^	0.93 (0.88, 0.97)^†^	0.97 (0.91, 1.03)	0.88 (0.83, 0.93)^#^
Refined grains	0.99 (0.97, 1.01)	0.99 (0.98, 1.01)	0.99 (0.98, 1.01)	1.00 (0.98, 1.02)
Fruit juice	0.94 (0.87, 1.01)	0.94 (0.88, 1.00)	0.96 (0.90, 1.04)	0.95 (0.86, 1.06)
Starchy vegetables	1.10 (0.99, 1.22)	1.04 (0.94, 1.14)	1.03 (0.95, 1.13)	1.16 (1.04, 1.29)^*^
Added sugar	1.01 (1.01, 1.02)^#^	1.01 (1.01, 1.01)^#^	1.01 (1.00, 1.01)^†^	1.01 (1.00, 1.01)^*^

OR, odds ratio; 95% CI, 95% confidence interval. ^*^P < 0.05, ^†^*P* < 0.01, ^#^*P* < 0.001.

Adjusted model was adjusted for age, sex, race/ethnicity, education level, marital status, family poverty-to-income ratio, smoking status, alcohol consumption, physical activity, BMI, total energy intake, hypertension, diabetes, and hyperlipidemia.

Regarding sensitivity analyses in Supplementary Table S2, the relationship between the quality and quantity of carbohydrate consumption and poor sleep patterns remained robust even after additional accounting for depression. Similarly, further adjusting for HEI-2015 scores did not materially change results. When participants with extreme energy intake were removed, the results remained consistent. Additionally, when additional adjustment was made for saturated fatty acids, total fat, and dietary fiber, higher consumption of low-quality carbohydrates and total daily carbohydrates was related to an elevated likelihood of unhealthy sleep, while higher intake of high-quality carbohydrates showed no such link. Similarly, even when incorporating dietary factors (saturated fatty acids, total fat, and dietary fiber) in the multivariate analysis, the participants with carbohydrate intake pattern 4 showed a lower likelihood of experiencing poor sleep patterns than those with carbohydrate intake pattern 1.

## Discussion

Using a cross-sectional study design, our research examined the correlation between the quantity and quality of carbohydrate consumption and sleep patterns. Our findings indicated that higher consumption of high-quality carbohydrates was linked to a lower likelihood of experiencing poor sleep patterns, whereas increased consumption of low-quality carbohydrates and total daily carbohydrates was associated with an elevated risk of poor sleep patterns. Additionally, we observed that individuals who had a diet high in high-quality carbohydrates and low in low-quality carbohydrates might be at a decreased risk for experiencing poor sleep patterns. Regarding the specific food composition, consuming more added sugar was linked to an increased probability of developing poor sleep patterns, whereas a diet rich in whole grains, fruits, or non-starchy vegetables was correlated with a decreased likelihood of experiencing poor sleep patterns.

Previous investigations have demonstrated the effect of different dietary patterns on sleep quality. Individuals who highly adhere to the Mediterranean diet are less likely to experience poor sleep quality, inadequate sleep duration, and excessive daytime sleepiness (25–27). Additionally, a cross-sectional study revealed that adhering to a Dietary Approaches to Stop Hypertension (DASH) diet was correlated with improved sleep quality (28). Another cross-sectional study indicated that individuals with higher HEI-2015 scores had a reduced risk of developing sleep disorders (22). Moreover, lower dietary quality has been linked to poorer sleep quality (29, 30). These studies lack a unique definition of a “healthy” diet; however, all dietary patterns share some common features, such as a high intake of plant-based foods like fruits and vegetables, whole grains and legumes, and unsaturated fatty acids and a low intake of processed foods and added sugars. Whole fruits, non-starchy vegetables, whole grains, and legumes in these healthy diets are considered high-quality carbohydrates, while added sugars are considered low-quality carbohydrates. Our study is the first to examine the correlation between the quality and quantity of daily carbohydrate intake and sleep. We observed that the consumption of high-quality carbohydrates was linked to a lower risk of poor sleep patterns, while there was a significant relationship between the consumption of low-quality carbohydrates and an elevated risk of poor sleep patterns. Besides, we also found a U-shaped association between the total daily quantity of carbohydrates and unhealthy sleep patterns. When the daily consumption of carbohydrates exceeds 15.9 servings, there is an increased risk of developing poor sleep patterns with an increase in carbohydrate intake. We speculate that the main reason may be an increase in total daily carbohydrate intake, which predominantly consists of low-quality carbohydrates. Consistent with previous research (31), our results also indicated that individuals experiencing poor sleep quality consume more carbohydrates than those with normal sleep quality. Consequently, low and especially high levels of carbohydrate consumption may be associated with an elevated risk of poor sleep patterns. In contrast, moderate carbohydrate consumption, about 15.9 servings per day, may be linked to a reduced risk of poor sleep patterns. This analysis highlights the importance of carbohydrates’ quality and quantity in mitigating sleep disorders. This discovery suggests that the risk of sleep disorders may be reduced by increasing the consumption of high-quality carbohydrates or reducing the consumption of low-quality carbohydrates.

A prior investigation revealed a positive connection between elevated intake of added sugars, starch, and refined grains and the risk of experiencing insomnia. In contrast, an increase in intake of dietary fiber, whole grains, non-juice fruits, and vegetables was linked to a decreased risk of insomnia (15). Subsequently, another study corroborated that consuming a higher amount of added sugars was related to an elevated risk of poor sleep quality (29). Nevertheless, both studies were confined to female participants, limiting the findings’ generalizability. Katagiri et al. (13) identified a significant correlation between low consumption of vegetables and high consumption of candy and noodles with poor sleep quality. Furthermore, high consumption of beverages, such as sugary drinks and fruit juice, has been linked to poor sleep quality (30). Both studies examined populations in China and Japan, respectively, while dietary habits vary between populations in the East and West. Our research specifically investigated the American population, encompassing individuals of various genders, races, and age groups. The findings revealed a positive relationship between high added sugar intake and an elevated risk of developing poor sleep patterns, as well as a negative correlation between increased consumption of whole grains, fruits, or non-starchy vegetables and a decreased likelihood of experiencing poor sleep patterns. Hence, we recommend increasing the consumption of whole grains, whole fruits, and non-starchy vegetables while restricting the intake of added sugars.

The potential pathways through which carbohydrates affect sleep are as follows. First and most important, carbohydrates have been shown to potentially affect sleep via the synthesis of tryptophan, serotonin, and melatonin (32). Research suggested that fruits, vegetables, and whole grains are abundant sources of tryptophan, a precursor to serotonin and melatonin, which must compete with other large neutral amino acids for passage through the blood–brain barrier. An increase in carbohydrate intake can promote the release of insulin, which facilitates the transfer of large neutral amino acids to the periphery for protein synthesis in muscles, allowing more tryptophan to penetrate the blood–brain barrier for the synthesis of melatonin and serotonin (32–34). Consuming a diet high in fruits and vegetables has been shown to increase the abundance of lactobacilli and bifidobacteria, while suppressing the growth of Bacteroides, Enterobacteriaceae, and Clostridium, ultimately leading to an improvement in gut microbiota composition (35, 36). Conversely, a diet rich in fructose may diminish the abundance of gut microbiota (37). The diversity and richness of gut microbiota have been linked to better sleep (38). Furthermore, vegetables, fruits, and legumes possess anti-inflammatory properties, whereas excessive sugar consumption promotes inflammation (39, 40). It has been demonstrated that an anti-inflammatory diet was correlated with enhanced sleep quality (41).

Several limitations of our study warrant discussion. While we have investigated the correlation between the quantity and quality of carbohydrate consumption and sleep, we did not examine the relationship between the timing of carbohydrate intake and sleep, especially the impact of carbohydrate intake at night on sleep quality. Additionally, carbohydrate intake and sleep issues in our study were based on self-reported data rather than objective measures, which was susceptible to memory bias. Furthermore, the relationship between the quantity and quality of carbohydrate intake and sleep patterns remains speculative, and causality cannot be established due to the inherent limitations of cross-sectional study designs. Finally, although we implemented multivariate analysis and sensitivity analyses, selective bias may still affect our results.

## Conclusion

In summary, a positive correlation was found between increased consumption of low-quality carbohydrates and an elevated risk of poor sleep patterns, while increased consumption of high-quality carbohydrates was linked to a decreased risk of developing poor sleep patterns. Further cohort studies or intervention studies are necessary to verify this association and investigate the potential of dietary changes in improving sleep quality.

## Data Availability

The datasets presented in this study can be found in online repositories. The names of the repository/repositories and accession number(s) can be found: All data are publicly available and can be accessed at the NHANES (https://www.cdc.gov/nchs/nhanes/index.htm).

## Footnotes

^1^ https://www.cdc.gov/nchs/nhanes/index.htm

^2^ https://wwwn.cdc.gov/nchs/nhanes/tutorials/
