# Supplementary material for: The relationship between carbohydrate intake and sleep patterns
Source: Front Nutr. 2024 Dec 4;11:1491999. doi: 10.3389/fnut.2024.1491999 (PMC11652137; doi:10.3389/fnut.2024.1491999)
Supplement: Supplementary file 1 [file Table_1.docx]

Supplementary table 1 Dietary Components of high- and low-quality carbohydrates.

| Carbohydrates | Food Patterns Equivalent Database (FPED) Unit representing 1 serving |
| --- | --- |
| High-quality carbohydrates |  |
| Whole grains | Ounce equivalent |
| Legumes | Cup equivalent |
| Whole fruit | Cup equivalent |
| Non-starchy vegetables | Cup equivalent |
| Low-quality carbohydrates |  |
| Refined grains | Ounce equivalent |
| Fruit juice | Cup equivalent |
| Starchy vegetables | Cup equivalent |
| Added sugars | Teaspoon equivalent |

Supplementary Table 2 Associations of daily carbohydrate consumption quantity and quality with sleep patterns in sensitivity analyses.

|  | Model III+HEI-2015 | Model III+Depression | Model III+dietary factors^&^ | Model III^%^ |
| --- | --- | --- | --- | --- |
|  | OR (95% CI) | OR (95% CI) | OR (95% CI) | OR (95% CI) |
| Carbohydrate intake patterns |  |  |  |  |
| Pattern 1 | Reference | Reference | Reference | Reference |
| Pattern 2 | 0.89 (0.75, 1.05) | 0.86 (0.72, 1.03) | 0.87 (0.73, 1.04) | 0.86 (0.72, 1.02) |
| Pattern 3 | 0.98 (0.84, 1.14) | 0.95 (0.81, 1.11) | 1.10 (0.92, 1.32) | 0.91 (0.78, 1.05) |
| Pattern 4 | 0.71 (0.62, 0.83)^#^ | 0.67 (0.58, 0.78)^#^ | 0.80 (0.67, 0.95)^*^ | 0.64 (0.55, 0.73)^#^ |
| P for trend | <0.001 | <0.001 | 0.049 | <0.001 |
| High-quality carbohydrate |  |  |  |  |
| T1 | Reference | Reference | Reference | Reference |
| T2 | 0.91 (0.78, 1.05) | 0.90 (0.77, 1.04) | 0.95 (0.82, 1.11) | 0.87 (0.76, 1.01) |
| T3 | 0.80 (0.67, 0.94)^*^ | 0.74 (0.65, 0.85)^#^ | 0.86 (0.71, 1.03) | 0.70 (0.61, 0.81)^#^ |
| P for trend | 0.010 | <0.001 | 0.090 | <0.001 |
| Low-quality carbohydrate |  |  |  |  |
| T1 | Reference | Reference | Reference | Reference |
| T2 | 1.10 (0.94, 1.28) | 1.14 (0.98, 1.34) | 1.09 (0.92, 1.28) | 1.17 (1.01, 1.36)^*^ |
| T3 | 1.27 (1.10, 1.47)^†^ | 1.37 (1.18, 1.60)^#^ | 1.32 (1.11, 1.56)^†^ | 1.41 (1.22, 1.64)^#^ |
| P for trend | 0.003 | <0.001 | 0.003 | <0.001 |
| Total carbohydrates |  |  |  |  |
| T1 | Reference | Reference | Reference | Reference |
| T2 | 1.11 (0.93, 1.32) | 1.14 (0.95, 1.37) | 1.12 (0.93, 1.35) | 1.15 (0.97, 1.38) |
| T3 | 1.22 (1.03, 1.46)^*^ | 1.30 (1.08, 1.56)^*^ | 1.29 (1.05, 1.59)^*^ | 1.34 (1.12, 1.60)^†^ |
| P for trend | 0.030 | 0.010 | 0.020 | 0.002 |

OR, odds ratio; 95% CI, 95% confidence interval. ^*^P<0.05, ^†^P<0.01, ^#^P<0.001.

Model III adjusted for age, sex, race/ethnicity, education level, marital status, family poverty-to-income ratio, smoking status, alcohol consumption, physical activity, BMI, total energy intake, hypertension, diabetes, and hyperlipidemia.

^&^Further adjusted for Model III+ dietary factors (saturated fatty acids, total fat and dietary fiber).

^%^We excluded participants with extreme energy intake levels below 500 kcal or exceeding 6000 kcal from the analysis.
