# Supplementary material for: The relationship between carbohydrate intake and sleep patterns
Source: Front Nutr. 2024 Dec 4;11:1491999. doi: 10.3389/fnut.2024.1491999 (PMC11652137; doi:10.3389/fnut.2024.1491999)
Supplement: Supplementary file 2 [file Image_1.pdf]

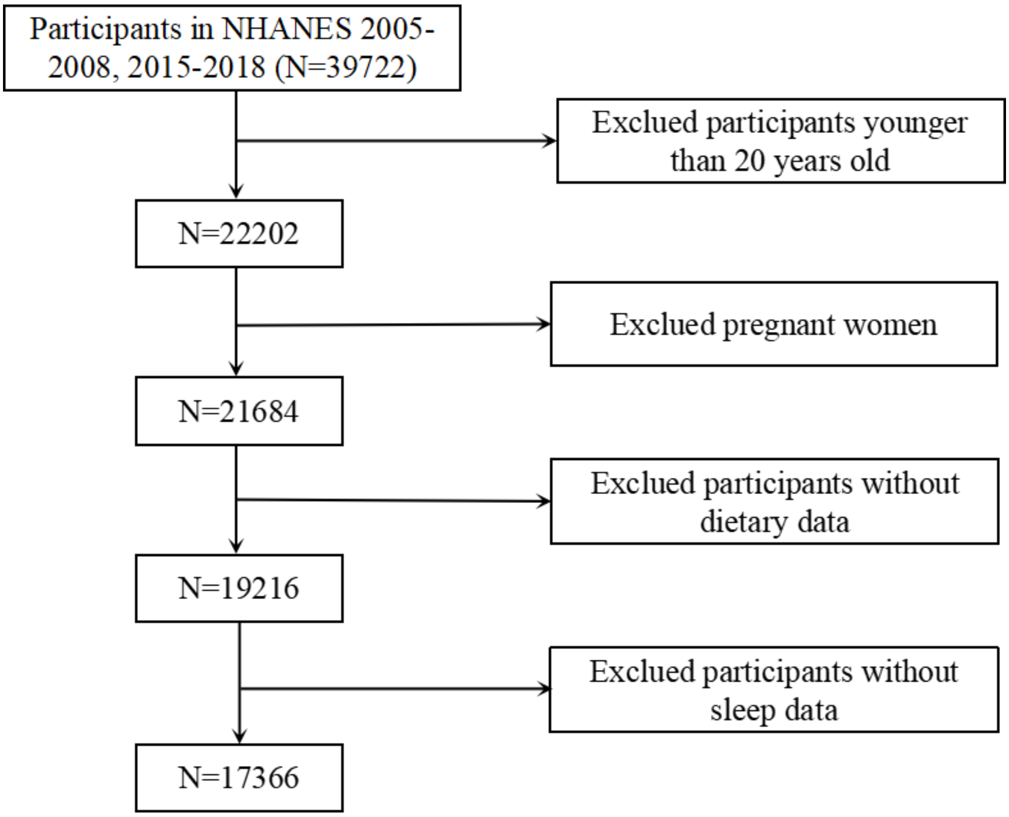

Supplement Figure 1 Flowchart of the sample selection from NHANES. NHANES, National Health and Nutrition Examination Survey.
